# A meta-analysis of cerebrospinal fluid visinin-like protein-1 in alzheimer’s disease patients relative to healthy controls and mild cognitive impairment patients

**DOI:** 10.17712/nsj.2017.2.20160557

**Published:** 2017-04

**Authors:** Xiaohui Hu, Yan Yang, Daokai Gong

**Affiliations:** *From the Department of Neurology (Hu, Gong), Jingzhou Central Hospital, Jingzhou Clinical Medical College, Yangtze University, Jingzhou, and from Hubei College of Chinese Medicine (Yang), Hubei Province, China*

## Abstract

**Objective:**

To compare cerebrospinal fluid visinin-like protein-1 (CSF VLP-1) in alzheimer’s disease (AD) with that in healthy controls and mild cognitive impairment (MCI) patients and find out possible sources of heterogeneity.

**Method:**

“Visinin-like protein-1” and “alzheimer’s disease” were employed to search “PubMed”, “Springer” and “Medline” databases until July 2016 and standard mean difference (Std.MD) was calculated. Besides, subgroup analysis and meta-regression were performed to explore the possible heterogeneity sources.

**Results:**

Seven studies involved 1151 participants were pooled. The CSF VLP-1 in AD patients was higher than that in healthy controls and MCI patients (pooled Std.MD=0.81, 95% CI: [0.47, 1.16], *p*<0.00001). As shown by subgroup analysis, population variations were one of heterogeneity sources. Meta-regression revealed that Hedges’s g of CSF VLP-1 was correlated with Std.MD of t-tau (r=0.560, *p*=0.006) and Ab42 (r=-0.386, *p*=0.013).

**Conclusion:**

The CSF VLP-1 in AD patients is higher than that in healthy controls and MCI patients. The changes of VLP-1 in AD patients relative to healthy controls and MCI patients is less pronounced than that of core biomarkers, such as Ab42, t-tau and p-tau. Population variations, increasing t-tau and decreasing Ab42 in AD patients relative to healthy controls and MCI patients were the main sources of heterogeneity.

Neuronal injury has been considered as the main pathological process of cognitive decline in alzheimer’s disease (AD).^1^ The main hallmark of AD, such as senile plaques and neurofibrillary tangles, which caused by the deposition of amyloid beta (Ab) and tau protein in brain, can result in neuronal injury.^2^ Decreasing amyloid beta42 (Ab42) levels and increasing total tau (t-tau) and phosphorylated tau (p-tau) levels in cerebrospinal fluid (CSF) have been regarded as the signature of AD patients.^3^ Furthermore, these core biomarkers are highly sensitive and specific to distinguish patients with cognitive impairment due to AD from healthy controls^4^ and other diseases, such as mild cognitive impairment (MCI),^5^ dementia with Lewy bodies,^4^,^6^ Parkinson’s diseases dementia,^4^,^6^ and progressive supranuclear palsy.^7^ Although these core CSF biomarkers, including Ab42, t-tau and p-tau have been used as CSF evidence to diagnose AD,^3^ it cannot represent the direct process of neuronal injury. Few biomarkers of neuronal injury have been revealed, such as neurofilament light protein and neurogranin, which respectively represent the loss of large-caliber myelinated axons and synapses.^8^ As a novel biomarker of neuronal injury, visinin-like protein-1 (VLP-1) is a neuronal specific calcium-sensor protein, which belongs to the calmodulin superfamily^9^ and participates in the fundamentally synaptic plasticity and memory formation.^10^ Many studies have been conducted to compare CSF VLP-1 levels in AD patients with that in healthy controls and MCI patients,^11^-^16^ but to date, the changes of CSF VLP-1 in AD patients relative to healthy controls and MCI patients are conflicting.^17^ Therefore, current meta-analysis was performed to compare CSF VLP-1 in AD patients with that in healthy controls and MCI patients. Furthermore, subgroup analysis and meta-regression were carried out to calculate the contributions of age, mini-mental state examination (MMSE) scores and levels of core CSF biomarkers in AD patients to the possible sources of heterogeneity.

## Methods

According to the proposal of the Meta-analysis of Observational Studies in Epidemiology (MOOSE),^18^ systematic searching databases of “PubMed”, “Springer”, and “Medline”, scanning references listed in articles were conducted to retrieve the published studies until July 2016 with language restriction as “English”. The following combined keywords, such as “visinin-like protein-1” and “alzheimer’s disease”, were utilized.

### Inclusion and exclusion criteria

Inclusion criteria were listed as follows: 1) the patients diagnosed with AD; 2) retrospective or prospective case-control study designs; 3) the CSF VLP-1 levels were detected. Furthermore, exclusion criteria included: 1) non-human studies, commentaries, reviews, meetings, and editorials or manuscripts unrelated to the research topic; 2) case reports and case series; 3) studies concerning children, adolescents and pregnant women; 4) unsuitable data were displayed.

### Data extraction and quality assessment

Two investigators reviewed the search results and selected articles to determine eligibility and extract study data. For assessing the study quality, the Newcastle-Ottawa Scale (NOS) criteria,^19^ which include the selection (0-4 scores), comparability (0-2 scores), and exposure (0-3 scores) categories (0 denoted noncompliance with any criteria, 9 denoted fulfillments of all criteria), were utilized. Studies were high quality methodology in accordance with NOS scores more than 6 scores.

### Statistical analysis

Heterogeneity was quantified with the I-squared (I^2^) statistic. For estimates of standard mean difference (Std.MD) and 95% confidence interval (CI) from individual studies, we adopted a weighted fixed-effect model when the heterogeneity tests *p*≥0.05. Accordingly, we adopted a random-effect model when the heterogeneity tests *p*<0.05. The Std.MD of CSF VLP-1, Ab42, t-tau, p-tau levels and MMSE scores among AD patients comparing with those among healthy controls and MCI patients were calculated by separate meta-analysis. Sensitivity analysis had been carried out with subgroup analysis, and funnel plots assessed publication bias. Additionally, heterogeneity, pooled Std.MD, sensitivity analysis, and funnel plots were calculated by Review Manager Version 5.3 (Copenhagen: The Nordic Cochrane Centre, The Cochrane Collaboration, 2014). Beyond that, separate regression of mean age and MMSE scores among AD patients, Std.MD of CSF Ab42, t-tau and p-tau levels as well as MMSE scores between AD patients and controls on Hedges’s g comparing the CSF VLP-1 in AD patients with controls were calculated by the method-of-moments technique. Meta-regression was performed by Comprehensive Meta-Analysis 2 (Biostat, Englewood, NJ, USA).

## Results

### Literature search strategy and characteristics of studies involved

**Figure 1:**
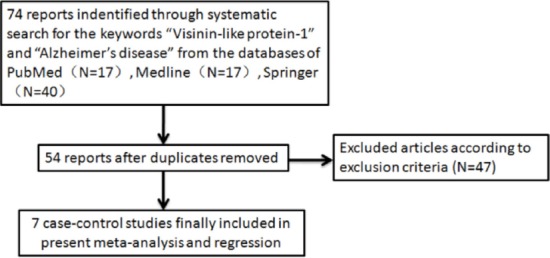
- The literature search strategy and 7 original studies were selected from the databases of “PubMed”,“Springer”, and “Medline” according to the inclusion and exclusion criteria

A total of 54 studies were identified after removing duplicate reports (N=20). According to the exclusion criteria, 7 case-control studies were identified after removing 47 reports. Details of literature search strategy were charted in **Figure 1**. According to NOS criteria,^19^ the total quality scores of 7 included studies^11^-^17^ indicated a high quality methodology of included original studies. General characteristics of included studies and participants were listed in **Table 1**. Extracted from 7 studies,^11^-^17^ 10 groups were pooled, in which it involved 629 AD patients and 522 participants including healthy controls and MCI patients. These studies were conducted in Netherlands, USA, Croatia, Poland and China. Enzyme linked immunosorbent assay was employed to detect CSF VLP-1 levels in 6 included groups, while microparticle-based immunoassay was adopted in the other 4 included groups. Additionally, the years of included studies ranged from 2008 to 2016 in the present meta-analysis.

**Table 1:** The general characteristics of involved studies.

Author Year	Nation	Mean age		Mean MMSE scores		Non-AD groups	CSF VLP-1 (ng/ml)		Sample Tec.	Number	
		AD	Non-AD	AD	Non-AD		AD	Non-AD		AD	Non-AD
Tarawneh 2015^11^	USA	73.6(8.7)	72.1(3.3)	26(3.7)	29(0.8)	Healthy	549(177.6)	396(66)	MBI	23	64
Mroczko 2016^12^	Poland	NR	NR	18(5.6)	29(1.5)	Healthy	107(63.7)	41(60)	ELISA	33	18
Mroczko 2016^12^	Poland	NR	NR	18(5.6)	26(3.2)	MCI	107(63.7)	70(25.7)	ELISA	33	15
Kester 2015^17^	Netherlands	65(4.8)	64(9.6)	22(3.4)	28(1.5)	Healthy	182(48.8)	168(53.7)	MBI	65	37
Kester 2015^17^	Netherlands	65(4.8)	68(4.8)	22(3.4)	27(1.5)	MCI	182(56)	192(73)	MBI	65	61
Babić Leko2016^13^	Croatia	72(8.2)	50(9.8)	20.1(4.6)	27.7(1.7)	Healthy	147.9(84.8)	65.5(49.3)	ELISA	109	9
Babić Leko2016^13^	Croatia	72(8.2)	68(9.8)	20.1(4.6)	24.9(2.9)	MCI	147.9(84.8)	98.7(80.3)	ELISA	109	43
Luo 2013^14^	China	68.1(7.6)	65.1(6)	12.1(3.9)	26.3(3.2)	Healthy	72.1(21.2)	43(9.5)	ELISA	61	40
Tarawneh 2011^15^	USA	74.9(8.1)	72.1(7.1)	25.3(3.8)	28.9(1.3)	Healthy	520(117)	396(149)	MBI	98	211
Lee 2008^16^	USA	67(6.8)	68.5(4.9)	23(4.1)	29.8(0.4)	Healthy	368(129)	245(113)	ELISA	33	24

Data were showed as mean (SD). MMSE - mini-mental state examination, AD - alzheimer’s disease, CSF - cerebrospinal fluid, VLP-1 - visinin-like protein 1, Tec - technique, MBI - microparticle-based immunoassay, ELISA - enzyme-linked immunosorbent assays, MCI - mild cognitive impairment, NR - not reported

### The CSF biomarkers levels, MMSE scores and age in AD patients

**Figure 2:**
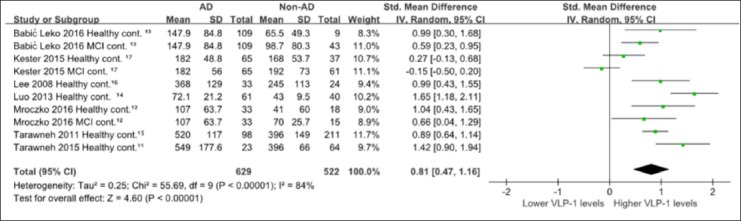
- The cerebrospinal fluid VLP-1 levels in AD patients are higher than that in non-AD participants including cont and MCI patients. VLP-1 - visinin-like protein-1, AD - alzheimer’s disease, cont - healthy control, MCI - mild cognitive impairment

When comparing CSF VLP-1 levels in AD patients with that in healthy controls and MCI patients, there was obvious heterogeneity (I^2^=84%) within the pooled 10 groups from 7 studies,^11^-^17^ CSF VLP-1 levels in AD patients were higher than that in healthy controls and MCI patients (pooled Std.MD=0.81, 95% CI: [0.47, 1.16], *p*<0.00001). The details were listed in **Figure 2**. Accordingly, CSF Ab42 levels in AD patients were lower than that in controls (pooled Std.MD=-0.97, 95% CI: [-1.34,-0.60], *p*<0.00001). The CSF t-tau levels in AD patients were higher than that in controls (pooled Std.MD=1.22, 95% CI: [0.80, 1.65], *p*<0.00001) and CSF p-tau levels in AD patients were higher than that in controls (pooled Std.MD=1.23, 95% CI: [0.86, 1.59], *p*<0.00001). The MMSE scores in AD patients were lower than that in controls (pooled Std.MD=-1.94, 95% CI: [-2.34,-1.53], *p*<0.00001). There was no age difference between AD patients and healthy controls and MCI patients (pooled Std.MD=0.37, 95% CI: [-0.07, 0.81], *p*=0.10). More details were listed in **Table 2**.

**Table 2:** Meta-analysis of cerebrospinal fluid Visinin-like protein 1 levels in AD patients relative to healthy controls and MCI patients.

Factors	Number of groups	weighted std.MD			Heterogeneity	
		Effect size	95% CI	*p*-value	I^2^(%)	*p*-value
VLP-1	10	0.81	0.47,1.16	<0.00001	84	0.00001
Ab_42_	10	-0.97	-1.34,-0.60	<0.00001	74	0.00001
T-tau	10	1.22	0.80,1.65	<0.00001	88	0.00001
P-tau	10	1.23	0.86,1.59	<0.00001	83	0.00001
MMSE scores	10	-1.94	-2.34,-1.53	<0.00001	85	0.00001
Age	8	0.37	-0.07,0.81	0.10	90	0.00001

Std.MD - standard mean differences, CSF - cerebrospinal fluid, Aβ_42_-amyloid beta42, T-tau - total tau, P-tau - phosphorylated tau, MMSE - mean mini-mental state examination scores, and mean age in alzheimer’s disease (AD) patients comparing with those in healthy controls and mild cognitive impairment (MCI) patients were calculated by inverse variance weighted model in turns

### Sensitivity analysis

According to the including conditions, such as, population variants, detection technique of the CSF VLP-1 levels, characteristics of control group, and Std.MD of MMSE scores comparing AD patients with healthy controls and MCI patients, sensitivity analysis had been carried out by subgroup analysis. When studies conducting from Netherlands, USA, Croatia and Poland were pooled, there was no (I^2^=0%) or low (I^2^=2 to 59%) heterogeneity. Beyond that, in the subgroup of sample technique, control group, Std.MD of MMSE scores, obvious heterogeneity were respectively observed in turns. Meanwhile, when studies with detection technique of CSF VLP-1 with MPI were pooled, there was no CSF VLP-1 difference between AD patients and participants including healthy controls and MCI patients (pooled Std.MD=0.59, 95% CI: [-0.03, 1.21], *p*=0.06). Furthermore, when CSF VLP-1 in AD patients were compared with that in MCI controls, no difference was found between these 2 groups (pooled Std.MD=0.34, 95% CI: [-0.22, 0.89], *p*=0.23). Besides, in order to estimate the stability and reliability of the pooled effect of CSF VLP-1, a fixed-effect model was adopted to compare AD patients and participants including healthy controls and MCI patients, which indicated that CSF VLP-1 levels in AD patients were higher than that in healthy controls and MCI patients (pooled Std.MD=0.73, 95% CI: [0.60, 0.87], *p*<0.00001).

### Publication bias

**Figure 3:**
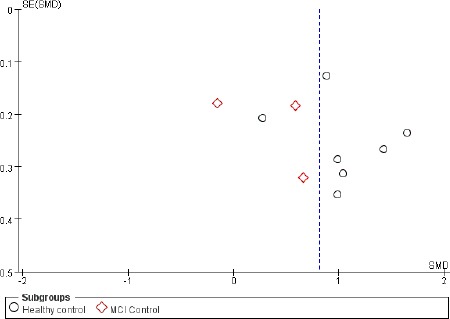
- The publication bias of included studies when comparing the cerebrospinal fluid visinin-like protein-1levels in alzheimer’s disease patients with that in healthy controls and MCI patients. MCI - mild cognitive impairment (MCI), SMD - standard mean difference, SE - standard error, MCI - mild cognitive impairment

In those 10 groups, publication bias was assessed with funnel plot (**Figure 3**). There was symmetrical distribution in the funnel plot, which suggested no risk of publication bias in the present meta-analysis.

### Meta-regression on increasing VLP-1 in AD patients relative to healthy controls and MCI patients

**Figure 4:**
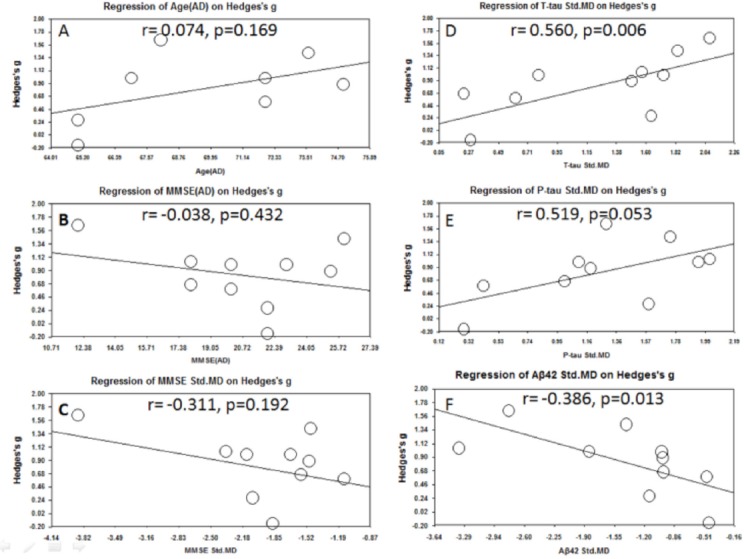
- Meta-regression on increasing cerebrospinal fluid (CSF) visinin-like protein-1(VLP-1) in alzheimer’s disease (**AD**) patients relative to healthy controls and mild cognitive impairment (MCI) patients. **A**) Separate meta-regressions of mean age, **B**) and mini-mental state examination(MMSE) of AD patients, **C**) as well as standard mean differences (Std.MD) of MMSE, **D**) total tau (T-tau), **E**) phosphorylated tau (P-tau), **F**) amyloid beta 42 (Ab42) in AD patients relative to healthy controls and MCI patients on Hedges’s g comparing the CSF VLP-1 levels in AD patients with that in healthy controls and MCI patients were performed

Age and MMSE scores among AD patients, Std.MD of MMSE scores, CSF t-tau, p-tau and Ab42 levels between AD patients and participants including healthy controls and MCI patients were pooled into the meta-regression model. Meanwhile, Hedges’s g comparing the CSF VLP-1 levels in AD patients with that in healthy controls and MCI patients was correlated with std.MD of t-tau (r=0.560, *p*=0.006) and Ab42 (r=-0.386, *p*=0.013). When comparing AD patients and participants including healthy controls and MCI patients, Hedges’s g of CSF VLP-1 had not significantly correlated with Std.MD of p-tau (r=0.519, *p*=0.053). In addition, no significant correlation was observed between Hedges’ g of CSF VLP-1 levels and mean age (r=0.074, *p*=0.169), MMSE scores (r=-0.038, *p*=0.432) among AD patients and MMSE scores Std.MD comparing the MMSE scores in AD patients with that in healthy controls and MCI patients (r=-0.311, *p*=0.192). Details of meta-regression were listed in **Figure 4**.

## Discussion

In this study, CSF core signature of AD was observed, such as reduced Ab42 and elevated t-tau and p-tau. Moreover, the current meta-analysis provided evidence that CSF VLP-1 levels in AD patients were higher than that in healthy controls and MCI patients. Meanwhile, the changes of VLP-1 were less pronounced than that of Ab42, t-tau, p-tau in CSF (**Table 2**). Obvious heterogeneity in original pooled studies weakened the persuasion of increasing CSF VLP-1 relative to healthy controls and MCI patients. However, subgroup analysis and meta-regression had revealed the main sources of heterogeneity.

Subgroup analysis was rationally used to estimate the influence of population variations, measurement technique of CSF VLP-1 levels, type of control groups and different degrees of Std.MD for MMSE scores on heterogeneity (**Table 3**). In the studies conducted in Netherlands, USA, Croatia and Poland, no or low heterogeneity was found. Since there were no reports discussing variations in CSF VLP-1 levels among different countries, the evidence that population variation was an influence factor of CSF VLP-1 levels (**Table 3**) was provided at first. However, sample detection technique, control groups and different degrees of Std.MD for MMSE scores had not contributed to heterogeneity sources. Besides, when comparing CSF VLP-1 levels in AD patients with that in healthy controls and MCI patients, the pooled effect size calculated with fixed-effect model was consistent with that calculated with random-effect model, which indicated a stable and reliable result. Furthermore, although CSF VLP-1 levels in AD patients were significantly higher than that in healthy controls, CSF VLP-1 levels in AD patients were displayed to not be different from MCI controls by subgroup analysis (**Table 3**). In the original included reports, higher CSF VLP-1 levels in AD patients than that in MCI controls were reported in 2 studies,^12^,^13^ while an opposite result was reported by Kester MI’s study.^17^ According to the contribution of population variations to one of heterogeneity sources and case-control study limitations, population variations and selective bias might be accounted for the inconsistent results when comparing CSF VLP-1 levels in AD patients with MCI controls at first. Besides, the effect size of CSF VLP-1 comparing AD patients with healthy controls was higher than that comparing AD patients with healthy controls and MCI patients (**Table 3**). This might be due to an increase of VLP-1 in MCI patients by as much as 10.7 pg/ml per year over time,^17^ which resulted in higher CSF VLP-1 levels in MCI controls than that in healthy controls. The increasing CSF VLP-1 levels in MCI controls relative to healthy participants maybe contribute to inconsistent results when comparing CSF VLP-1 levels in AD patients with that in healthy controls and MCI patients.

**Table 3:** Sensitivity analysis of the cerebrospinal fluid Visinin-like protein-1 (VLP-1) in Alzheimer’s disease patients relative to healthy controls and mild cognitive impairment patients.

Including condition	Number of groups	Weighted standard mean difference			Heterogeneity	
		Effect size	95% CI	*P*	I^2^	*P*
*Population variants*						
Netherlands	2	0.03	-0.24, 0.29	0.83	59%	0.12
USA	3	0.99	0.78, 1.19	<0.00001	39%	0.19
Croatia	2	0.67	0.35,0.99	<0.0001	2%	0.31
Poland	2	0.86	0.42,1.29	0.0001	0%	0.40
China	1	1.65	1.18,2.11	<0.00001	NA	NA
*Sample tec.*						
ELISA	6	0.99	0.63,1.34	<0.00001	63%	0.02
MBI	4	0.59	-0.03,1.21	0.06	91%	<0.00001
*Type of cont. group*						
Healthy control	7	1.02	0.68,1.36	<0.00001	74%	0.0008
MCI control	3	0.34	-0.22,0.89	0.23	80%	0.006
*Standard mean difference of MMSE scores*						
-1 to -2	6	0.71	0.27,1.15	0.002	85%	<0.00001
-2 to -3	3	0.73	0.20,1.26	0.0007	68%	0.04
More than -3	1	1.65	1.18,2.11	<0.00001	NA	NA
Fixed-effect model	10	0.73	0.60,0.87	<0.00001	84%	<0.00001

Sensitivity analysis had been carried out by subgroup analyses according to the including conditions which were the population variants, detection technique to VLP-1, standard mean difference (Std.MD) of Mini-Mental State Examination (MMSE) scores comparing AD with controls, and the fixed effect model in turns. NA - Not Applicable. MBI - Microparticle-based immunoassay, ELISA - Enzyme-linked immunosorbent assays.

Although the effect size of CSF VLP-1 levels in AD patients comparing with that in healthy controls and MCI patients was less pronounced than that of core CSF biomarkers of AD, such as Ab42, t-tau and p-tau (**Table 2**), the role of CSF VLP-1 as a biomarker of neuronal injury in AD has been supported in some degree by current study.^9^,^11^,^20^ Stejskal D et al^20^ had confirmed the role of increasing serum VLP-1 in estimating neuronal injury after ischemic stroke. Moreover, whole-brain and regional atrophy which derived from neuronal injury/loss had been correlated with VLP-1 and tau phosphorylated at threonine 181 (P-tau 181) levels in CSF.^11^ Besides, due to positive correlation between obvious neuronal loss in AD patients’ hippocampus and cerebral cortex and cognitive decline, CSF VLP-1 might be utilized to assess AD progression as a biomarker of neuronal injury.^21^ Although a negative correlation between CSF VLP-1 levels and MMSE scores among AD patients was reported by Lee JM et al’s study,^16^ no correlation between VLP-1 and MMSE scores was supported by 2 included studies.^12^,^14^ It might be explained that VLP-1 represented a valuable biomarker for assessing the progression at the early stage rather than the late stage of AD. The reason was that in Luo X et al and Mroczko B et al’s studies (mean scores: 18.0), MMSE scores of AD patients^12^,^14^ were lower than those in Lee JM’s study^16^ (mean scores: 23.0), which maybe indicated an earlier stage of AD in Lee JM’s study^16^ than that in Luo X et al and Mroczko B et al’s studies.^12^,^14^ Moreover, the role of CSF VLP-1 in predicting global cognitive decline rates had been further supported by a longitudinal follow-up study for early AD patients.^22^

Some studies indicated that the association between VLP-1 and other core CSF biomarkers, such as Ab42 and t-tau was noncommittal.^12^-^14^,^16^ In other words, a positive correlation between VLP-1 and t-tau had been reported by 3 studies,^13^,^14^,^16^ however no correlation between VLP-1 and t-tau in CSF had been revealed by Mroczko B et al.^12^ Besides, although a negative correlation between VLP-1 and Ab42 had been found by Babić Leko M et al,^13^ no correlation was shown in another 2 studies.^12^,^16^ As far as we know, this was the first meta-regression comparing CSF VLP-1 in AD patients with that in healthy controls and MCI patients. The present meta-regression had revealed that VLP-1 in AD was significantly correlated with t-tau (**Figure 4D**) and Ab42 levels (**Figure 4F**) in CSF, which indicated the increasing t-tau and decreasing Ab42 in CSF of AD patients relative to healthy controls and MCI patients contributed to some sources of heterogeneity when comparing CSF VLP-1 in AD patients with that in healthy controls and MCI patients. Although a positive correlation between CSF VLP-1 and p-tau had been strongly proved by 4 included studies,^12^-^14^,^16^ the present meta-regression showed a trend of positive correlation between CSF VLP-1 and p-tau without significance (*p*=0.053, **Figure 4E**). Nevertheless, VLP-1 had pathologically referred to molecular mechanisms of AD by correlation with amyloid precursor protein expression, which led to Ab deposition and neurodegeneration.^23^,^24^ Furthermore, positive correlation between VLP-1 levels and amyloid load in the brain had been revealed by a study of amyloid neuroimaging in vivo.^22^ Therefore, increasing t-tau and decreasing Ab^42^ related to CSF VLP-1 levels in CSF of AD patients relative to healthy controls and MCI patients. Increasing CSF p-tau levels in AD patients relative to healthy controls and MCI patients may be a possible source of heterogeneity. More studies were still needed to confirm the correlation between CSF VLP-1 and p-tau.

### There are several limitations in current study

First, since all including original studies were observational research, the selective bias was unavoidable. Second, although CSF VLP-1 levels were higher in AD patients than that in healthy controls and MCI patients (**Figure 2**), there was no difference of CSF VLP-1 levels between AD and MCI controls (**Table 3**). Third, when the quality of included studies was assessed by NOS criteria, age (**Table 2**) and APOE genotype^11^,^16^ differences between AD patients and participants including healthy controls and MCI patients might contribute to the confounding bias and influence the comparability.^19^

In conclusion, CSF VLP-1 levels in AD patients were higher than that in healthy controls and MCI patients. Meanwhile, the changes of CSF VLP-1 in AD patients relative to healthy controls and MCI patients were less pronounced than that of core CSF biomarkers, including Ab42, t-tau and p-tau. As shown by subgroup analysis and meta-regression, population variations, increasing t-tau and decreasing Ab42 in CSF of AD patients relative to healthy controls and MCI patients were the main sources of heterogeneity.
